# The new framework of innovation biosphere for analysing innovation policies facing COVID-19 grand challenge

**DOI:** 10.1186/s12961-024-01148-0

**Published:** 2024-06-13

**Authors:** Shohreh Nasri, Mehdi Fatemi, Najmeh Nazeri, Sepehr Ghazinoory

**Affiliations:** 1 Department of Science & Research Policy, National Research Institute for Science Policy, Tehran, Iran; 2 Department of Information Technology Management, Tarbiat Modares University, Tehran, Iran; 3 University of Tehran, Tehran, Iran

**Keywords:** Innovation biosphere, Grand challenges, COVID-19, Innovation policy, Global innovation, Mission-oriented innovation policy

## Abstract

**Background:**

Facing global grand challenges such as coronavirus disease 2019 (COVID-19) require the participation of various actors in different sectors and systematically directing their innovative efforts. Considering the complexity, non-linear dynamics, and global extent of the COVID-19 challenge, developing and applying a multi-level, resilient, and systematic innovative framework is vital. Therefore, this study aims to apply the “innovation biosphere” framework inspired by ecological studies for examining and analysing the management dimensions of COVID-19.

**Methods:**

In this research, based on a deductive-inductive approach, the case study methodology is used. In accordance with this strategy, the innovation biosphere metaphor is considered as the basic framework (deductive approach) and subsequently the grand challenge of COVID-19 (inductive approach) is analysed at three levels: micro, meso and macro.

**Results:**

The research findings verify the correspondence between what happened in the management of COVID-19 and the proposed framework of innovation biosphere. In other words, the findings of the research show that the effect of global cooperation, role-playing and co-evolution of different actors and subsystems in facing the grand challenge of COVID-19 under an ecosystemic and eco-innovation approach has been evident. These events subsequently led to the cessation of the pandemic after about four years.

**Conclusions:**

The main policy implications include the role of self-organization, the capability of global value networks, mission orientation, and co-evolution between actors as the contributions of innovation biosphere framework for managing grand health challenges, and global cohesion, oligopoly market, supporting local innovations, the critical role of basic research, and deregulation as the contributions of the COVID-19 case study for enhancing the innovation biosphere metaphor.

## Introduction

Given the rapid advances in global science and technology, nobody imagined that the coronavirus disease 2019 (COVID-19) pandemic would have such prolonged and severe effects. However, by May 2023, approximately 688 million people were infected with this virus, of which more than 6 million lost their lives.^1^ Finally, on 5 May 2023, the WHO announced the end of the pandemic after 4 years [64].

The global mobilization of science, technology and innovation (STI) played a fundamental role in improving the understanding of societies regarding COVID-19 and developing vaccines and therapeutic and diagnostic methods [58]. The public and private sectors invested billions of dollars worldwide, forming an unprecedented international collaboration. In other words, the COVID-19 crisis has significantly affected science, technology and innovation policies and the previous trends in applying science and technology, with a remarkable increase in free access to data and publications and the application of digital devices as examples. Therefore, in the long term, science, technology and innovation policy responses to COVID-19 will lead to an economic transition towards sustainable, resilient and inclusive futures [53, 56]. Although COVID-19 affected all countries indiscriminately [69], its long-term consequences have widened the economic and social gap between the Global North and South. In other words, COVID-19 caused interconnected grand challenges in different economic–social systems, which led to developing cross-border dynamics [20]. Challenging the profit-oriented global paradigm, it also revolutionized the typical pattern of employing science, technology and innovation policies, that is, the emergence of COVID-19 highlighted the consequences of neoliberal approaches to science and technology, including the exclusive and unfair patent regime, the loss of investments in public health and the expansion of new illegal frontiers that endanger people and the planet [3]. Therefore, although the pandemic was tragic, resulting in many human and financial losses, it also brought positive impacts, including the formation of global innovation networks and the development of an open innovation approach to solving this grand challenge [18, 54, 60].

Although innovation is thought to result from collaboration and networking – mainly through “open innovation” as the most common paradigm – the networks’ scope is usually limited to firms, sectors or national boundaries. There are limited examples of extensive international collaborations for innovation development. Despite the existence of global grand challenges (e.g. the spread of infectious diseases, poverty, air pollution, climate change, global warming, earthquakes and lack of fresh water), there is still no wide global network to face and manage such challenges. In addition, concepts such as Problem-oriented Innovation System (PIS) [24] and Mission-oriented Innovation System (MIS) [34], which have a challenge-based approach, mainly focus on the national level. In other words, the main concepts that have modelled the cooperation of different elements for the creation and development of an innovation are limited to the following categories: models focussed on organizational boundaries (such as the linear model of innovation or the organizational innovation system), models focussed on a specific industry or technology development (such as technological innovation system, sectoral innovation system, innovation ecosystem, etc.) [7, 12]; and models focussed on local boundaries (regional innovation system or national innovation system) [24]. However, the coronavirus pandemic provided an opportunity to overlook many restrictions (e.g. patent restriction policies), publish research quickly and temporarily ignore some ethical codes to “predict and treat COVID-19 and save human lives” as a mutual goal. Although scholars have previously proposed frameworks (e.g. global innovation systems [9]), no applied research has employed them due to the lack of suitable case studies.

Furthermore, in the last two decades, the innovation literature has tried to reach a better understanding of the innovation process by using biological and especially ecological metaphors. Despite developing metaphors such as innovation ecosystem [1], business ecosystem [48], innovation ecotone [25], and innovation biome [26] at the micro, meso, and macro levels, there is a research gap in theorizing the innovation biosphere with a transnational approach.

Therefore, the central premise of this study is that the policy response to COVID-19 has been similar to the development and evolution of a natural biosphere, given the development of scientific and technological collaborations in a global network with the characteristics of a complex adaptive system. However, corona epidemic management has yet to be analysed with an ecological lens. Therefore, this article first explains the identified gap in innovation studies while analysing the COVID-19 pandemic as a grand challenge. Then, using the ecological metaphor of the biosphere, it defines the innovation biosphere and its characteristics, and finally, matches the metaphor with what happened during the pandemic to resolve the metaphor’s theoretical flaws and present practical suggestions for policy reforms in cases similar to COVID-19.

## Theoretical foundations

### COVID-19 as a global grand challenge

In general, different characteristics have been presented in the literature for grand challenges. For example, we can mention features such as uncertainty, complexity, non-linear dynamics and multiple interactions [28]. They can be classified under five main categories: (a) they are complex and require “many interactions, immediate understanding, and non-linear dynamics”; (b) they bring “fundamental ambiguities” to the institutions in terms of planning for an uncertain future; (c) as “evaluative” and value-creating issues that are subject to interpretations and reconfigurations by multiple parties over time, they affect multiple value criteria and have the possibility of creating new concerns if resolved; (d) they affect many people given their widespread prevalence; and (e) their management requires coordinated and systematic innovative efforts among diverse institutions at different levels. Naturally, grand challenges arising at a transnational level will be more complex, with global innovation collaborations as their prominent feature (Scwoon et al., 2022; [5, 21, 59].

**Fig. 1:**
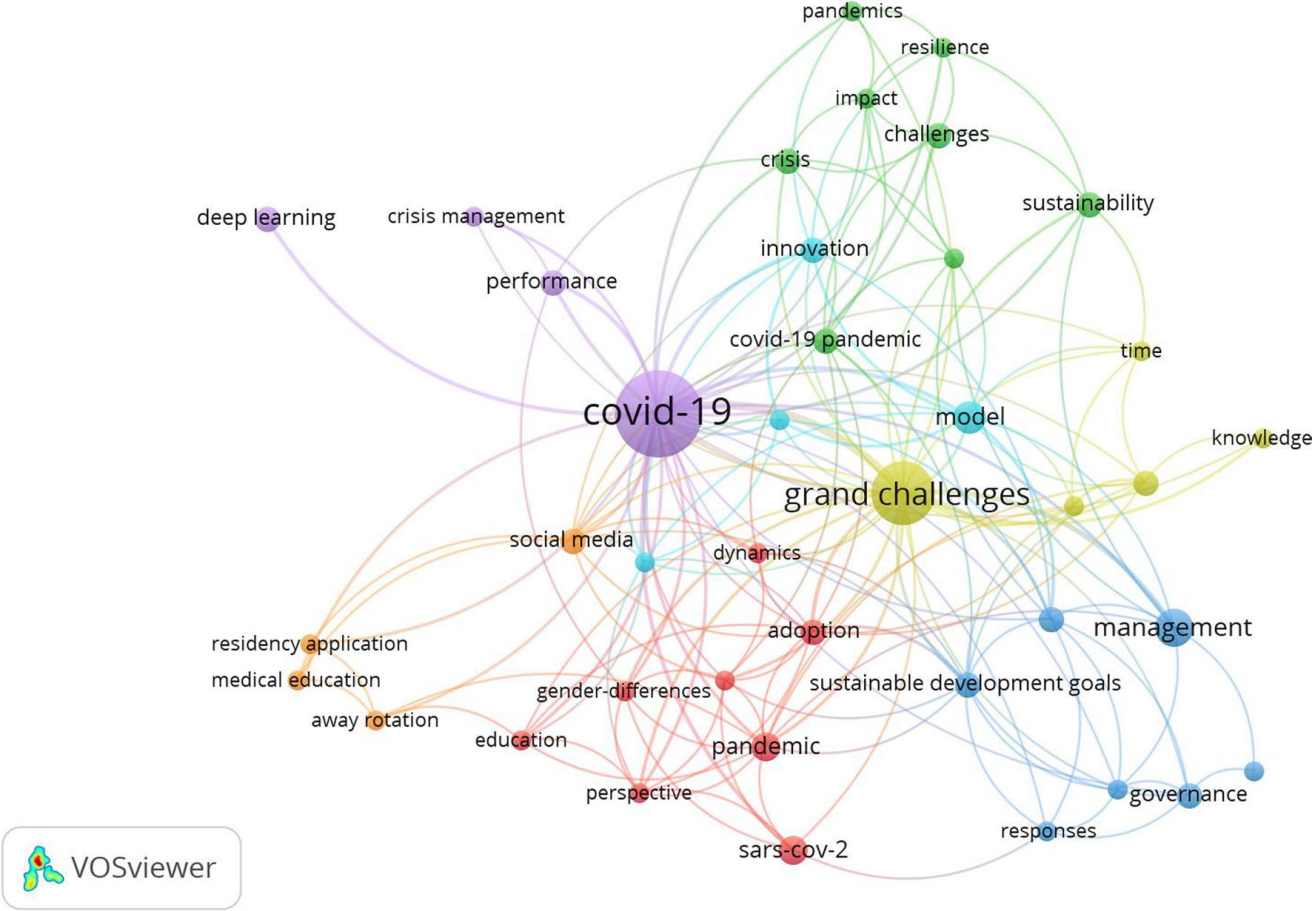
Co-occurrence of keywords in the “COVID-19 as a grand challenge” search string according to the Web of Science (WOS) dataset (To draw this graph, the search string “COVID-19 + Grand challenge” was used, and on the basis of the data entered into VOS viewer (including 62 articles), several main clusters were identified, which are shown in the Fig. 1)

The features mentioned above are very similar to the features of the COVID-19 challenge; therefore, some scholars have introduced COVID-19 as a grand challenge on a global scale (e.g. Bacq et al. [4], Bertello et al. [5], Howard-Grenville [29], Kost [30] and Schwoon et al. [59]). Management of a grand challenge such as COVID-19 requires the participation of a diverse range of actors in various sectors to achieve the expected scientific and technological advances. In addition, as a grand challenge on a global scale, the COVID-19 pandemic required the collective, coordinated and collaborative efforts of various actors and particular innovation policies [5]. Compared with traditional and insular policies, such policies present a comprehensive and integrated package of government interventions according to the innovation cycle to achieve ambitious goals by coordinating research, technology and innovation policies and integrating supply-side and demand-side policy tools with an interdisciplinary approach [53]. Figure 1 shows the co-occurrence of keywords in the “COVID-19 as a grand challenge” search string.

The COVID-19 grand challenge generally indicated the need to develop transnational innovation thinking and practices beyond national governance and move towards a global innovation system (biosphere). In addition, it boosted new trends in technology and global competition that challenged the innovation system theory and the traditional approach to formulating innovation policy [41]. Therefore, the need to introduce a new framework to face grand global challenges such as the COVID-19 pandemic is more evident than ever. This article uses a metaphorical approach to introduce the proposed framework by presenting grand challenges as complex phenomena and highlighting the role of analogies and metaphors in understanding these phenomena [19]. Accordingly, the following section introduces the innovation biosphere as a framework for analysing COVID-19 grand challenge through metaphors from biological and ecological studies.

### Conceptualization of innovation biosphere

The growing spatial complexity of innovation processes and the increasing blurring of geographical boundaries have challenged traditional and delimited systemic approaches (e.g. regional, local and national innovation systems). Some pioneering scholars criticize the emphasis on defined borders and the efforts of countries to keep research and development (R&D) secrets as firms are moving towards alliances with other firms in other countries to share part of the increasing costs of R&D, design, engineering and production and overcome domestic obstacles caused by government intervention [50]. This trend has become prominent in solving global grand challenges, with examples in the 20th and the beginning of the 21st century in the world economy and trade, subsequently leading to the development of numerous innovations on a global scale. Therefore, global innovation scholars believe that traditional approaches need changes to be applicable globally and internationally [9].

**Fig. 2:**
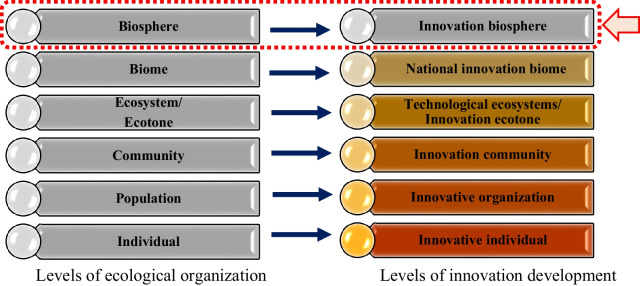
Correspondence between the levels of ecological organization and levels of innovation development [26]

Furthermore, the complexity of global innovation leads to the incapability of current metaphors (e.g. system and cluster) in explaining global dynamics (in terms of the complexity of the environment, interactions and structure); therefore, an alternative metaphor clarifies the dynamics of global interactions more precisely [41, 46, 50]. In this regard, given the remarkable similarity of innovation and ecological environments, ecological metaphors in general, which are welcomed by many scholars in the fields of management, business and innovation [48] [24], and the innovation biosphere in particular as an ecological and metaphorical mapping – which is the proposed macro-metaphor^2^ in the present study – have a unique capacity in developing a dynamic framework for conceptualizing mission-oriented global innovation (Fig. 2).

Accordingly, in light of the ecological insights of the natural biosphere,^3^ this research seeks to use the metaphor of the innovation biosphere to investigate and analyse the grand global challenge of COVID-19. Before conceptualizing the innovation biosphere, examining the general characteristics of innovation development at the international level is crucial.

On the basis of the literature review, the components of the global innovation process can be divided into three levels according to their focus: (1) the micro level focussed on actors, (2) the meso level focussed on the subsystems, and (3) the macro level focussed on the system and process (macro level). Figure 2 shows the general characteristics, components and types of actors for developing global innovation according to the category.

At the micro level, global actors refer to the types of general and specific actors in the global innovation value network. In more precise terms, the value network of the global innovation system [2, 10, 15, 45] is created under the influence of the interactions of specific and general global actors and ultimately leads to the creation of distinctive value or innovation. General actors (e.g. regulatory and legislative institutions, universities and entrepreneurs) are at the frontline of the global innovation value chain, directly forming global innovation [2, 9, 66]; specific actors (e.g. multinational firms) include system mediator actors who act as facilitators of network relations in the global system and have the ability to play a crucial role in several subsystems at the same time [36, 51, 66, 72]; the leader actor (e.g. international institutions) governs the global system according to its power and influence [2, 10, 11]; and the champion actor refers to influential and prominent individuals with natural or legal personalities (mostly natural) who desire to realize a particular global innovation for various reasons and motivations [13].

At the meso level, global innovation subsystems are various innovation biomes, ecosystems and ecotones of the global system, with several actors interacting under an institutional environment and specific resources to create value in the global innovation value network. The boundaries of an innovation subsystem are flexible and broad and can be national, regional, functional or technological. The institutional environment governing an innovation subsystem shapes the role, added value, value creation, interactions, risk and dependence of the actors of the subsystem [36, 74]; the resources of an innovation subsystem include all the tangible and intangible resources that cause the competitive advantage of that subsystem in the global system [13, 40, 72]; and finally, the interactions between the actors of a subsystem [9, 15, 61, 72] are relationships that mainly focus on collaboration, co-evolution and competition [37, 52, 62].

**Fig. 3:**
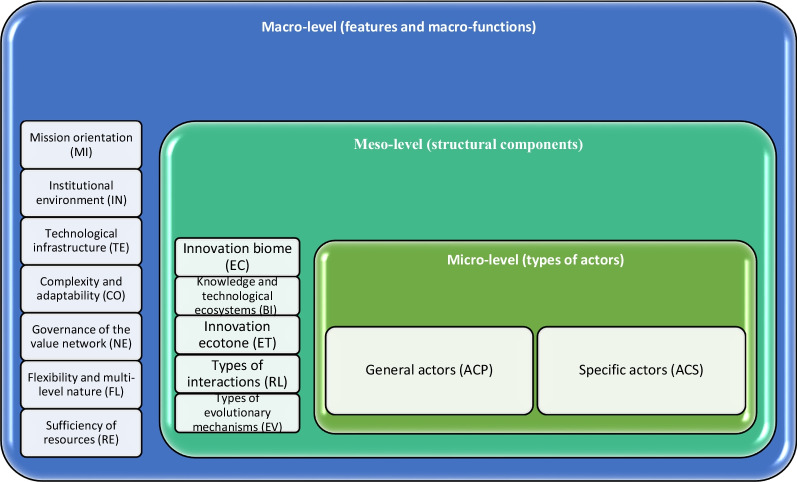
Characteristics, components and types of actors for the development of global innovation (compiled by authors; abbreviation codes shown in the figure are used in the findings section)

At the macro level, the global innovation system is a complex and adaptive [15, 52], mission-oriented and evolving [9, 10] value network with flexible and multi-level boundaries [37, 66, 74] that brings together general or specific actors with resources, institutional environment and unique interactions from diverse subsystems under one or more solid technological infrastructures [2, 39, 52]. This value network is mission oriented, formed to achieve innovation in a specific field that requires global participation (e.g. combat COVID-19). The global innovation system is complex [15, 52], as it operates as a complex self-organizing and adaptive system in conditions of uncertainty. It is evolving through various life cycle stages [55, 62, 66] and continues to operate on the basis of a specific technological infrastructure [2, 39, 52]. Finally, the global innovation system is flexible as it has multiple relationships from multiple subsystems to individuals and organizations in addition to systemic relationships with other global systems at lower levels (e.g. national and regional levels) [9, 37, 66, 74]. Figure 3 demonstrates the conceptual framework of the innovation biosphere according to the features of the global innovation process .

## Research methodology

**Fig. 4:**
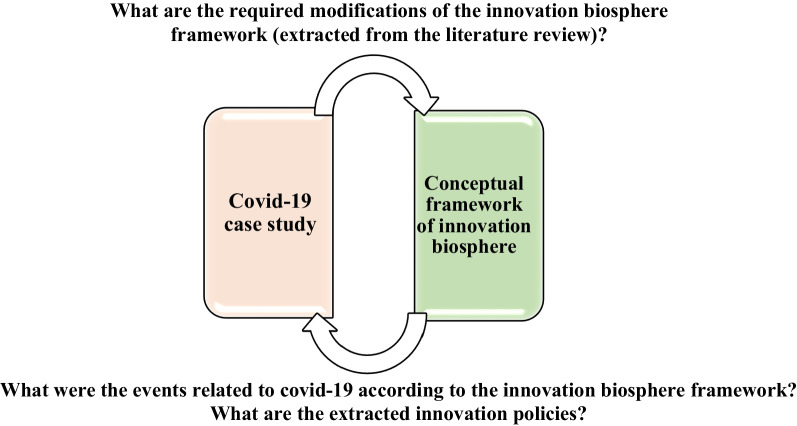
Deductive–inductive approach of the research

There are three categories of research approaches: deductive, inductive and deductive–inductive. This research considers the conceptual metaphor of the innovation biosphere as the fundamental framework (deductive approach) and accordingly collects textual data related to COVID-19 (inductive approach). Criticizing an inductive-only approach, multi-grounded theory (MGT) uses a combined deductive–inductive approach to clarify and generalize conceptual frameworks [23, 27]. Figure 4 demonstrates the deductive–inductive approach of this research.

**Fig. 5:**
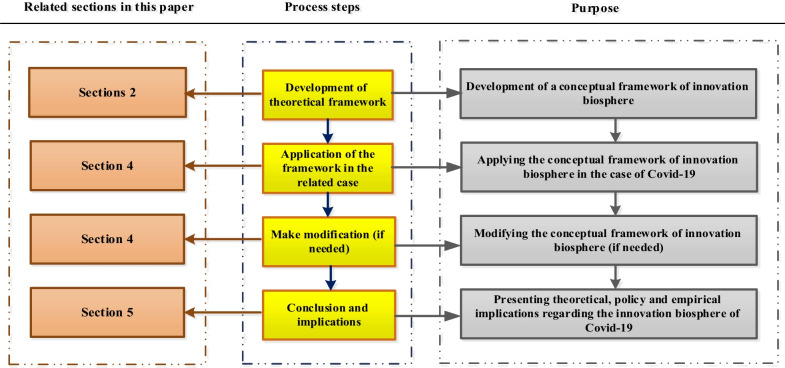
Research methodology and article structure

The case study strategy is employed, as it suits the deductive–inductive approach [73] to adapt the proposed metaphor of the innovation biosphere to what happened during the coronavirus pandemic, resolving the theoretical flaws of this proposed metaphor and ensuring its generalizability. Figure 5 illustrates the research methodology [73] and the structure of the article.

## Examining the COVID-19 grand challenge according to the innovation biosphere framework

The macro-conceptualization of the innovation biosphere metaphor is based on the central premise that innovative solutions to global challenges at national and international levels require adopting a global and mission-oriented approach, both in terms of the intervention of different actors and in terms of paying attention to the capacities of the global environment. In this regard, the current research hypothesizes that “the innovation biosphere metaphor can have experimental implications, especially in terms of identifying and mapping metaphorical examples of actors and related global environments to facilitate the formation of global collaboration for innovatively solving this grand challenge with an ecological-evolutionary approach”. Thus, activities towards facing the grand challenge of COVID-19 were at three macro, meso, and micro levels according to the conceptual framework of the research (the proposed metaphor).

### Micro level

The micro level focusses on the activities of various actors in the COVID-19 innovation biosphere. The main actors at the micro level can be classified into two general categories: general actors (ACP) (including all vertical and horizontal value chain actors in the health field) and specific actors (ACS) (including the leader actor, systemic intermediary actor and fundamental actor in the management of COVID-19). Given their generality, general actors include all actors involved in the global health value chain; public actors such as healthcare workers, public health authorities and agencies; researchers and scientists in various fields (e.g. molecular diagnosis, rapid diagnosis) [35]; data analysts and AI developers in the field of health [44, 65]; developers of digital technologies to reduce physical contacts and maintain social distance [71]; entrepreneurs; social activists designing and manufacturing medical devices (e.g. Pfizer, Moderna and AstraZeneca), medical masks (e.g. 3M, Honeywell and DuPont; Kimberly-Clark and Medline), testing devices (e.g. Hoffmann-La Roche AG and Abbott Laboratories and Thermo Fisher Scientific), air conditioning systems (e.g. Carrier and Trane) and sanitizers (e.g. Clorox and Lysol); small and medium manufacturers; and health agents (CHA) [70].

The specific actors of the COVID-19 innovation biosphere can be divided according to the conceptual framework into leader, umbrella, fundamental and system mediator actors. The WHO explicitly stated the need for a COVID-19 vaccine; the National Institute of Health accelerated the development and support of research on various treatments and the COVID-19 vaccine and coordinated various research platforms [14]; the United Nations collected and shared data related to COVID-19 [6, 33]; and the World Trade Organization provided medical supplies and reduced the economic effects of the COVID-19 disease [68]. These actors were the leaders of the COVID-19 innovation biosphere.

Multinational firms such as Pfizer (research and development of a COVID-19 vaccine in 2020), Johnson & Johnson (production of a single-dose COVID-19 vaccine), Moderna (in collaboration with National Institute of Allergy and Infectious Diseases (NIAID) and Biomedical Advanced Research and Development Authority (BARDA) in the field of innovation and development of COVID-19), International Business Machines (IBM) [development of technologies such as artificial intelligence (AI) and medical data analysis for disease control and rapid diagnosis of COVID-19], Amazon (providing online health and medical services and medical equipment), AstraZeneca (COVID-19 vaccine development and production in collaboration with Oxford University in the production of raw materials needed for vaccines and drugs in disease management), GSK (GlaxoSmithKline) (in vaccine development and production in collaboration with Sanofi), Roche (development and production of COVID-19 diagnostic tests in collaboration with Regeneron), Siemens Healthineers (development of COVID-19 diagnosis and treatment technologies including imaging technology and diagnostic tests), Novartis (development of drugs to manage the side effects of COVID-19 and drugs for treatment) and so on, have played a role in the innovation biosphere of COVID-19 as systemic mediating actors.

Champion actors with technical and scientific capabilities, leadership skills, international work experience, teamwork spirit, system thinking and experience in various fields of global innovation were Microsoft through the Bill and Melinda Gates Foundation (biosphere-level goals) and Tesla and SpaceX (production of medical equipment and development of digital technologies). Finally, fundamental actors with the ability to influence globally through providing financial resources and tax support for global innovation projects, establishing international collaborations, providing technical and specialized assistance and establishing new global innovation firms include the United States Food and Drug Administration (FDA) (Brace et al. 2022), Amazon (providing financial and technical aid to weaker countries), the Asian Infrastructure Investment Bank and the United Nations Foundation.^4^

### Meso level

Given the environmental dynamics and the variety of variants and mutations of the COVID-19 virus, paying attention to the actors’ evolution, interactions and co-evolution is vital. In line with the multiple mutations of the virus, the actors fighting against it co-evolved to be able to deal with its spread in the world effectively. The adaptation and evolution of online health businesses given the new global business environment (environmental adaptation evolution) (EV), the production of effective vaccines by various pharmaceutical companies (innovation-oriented evolution) (EV) [67], the presence of large pharmaceutical companies (e.g. Pfizer and AstraZeneca) in various countries and the evolution of host pharmaceutical companies due to interaction with them (performance improvement evolution) (EV) are the examples of actors’ evolution (EV) in the COVID-19 innovation biosphere. In addition, the competition between different vaccine manufacturers (e.g. Pfizer, Sinopharm and AstraZeneca) and predation relationships between actors producing or distributing illegal vaccines or drugs and supervisory and security institutions are instances of co-evolution in the biosphere (EV).

From an environmental point of view, it is also essential to pay attention to the mapping and analysis of the environment of the COVID-19 innovation biosphere as geographical, technological, institutional and functional coordinates that govern the evolution of global actors in dealing with COVID-19. The environment determines how actors act in different COVID-19 innovation biomes (e.g. Iran’s innovation biome with a unique formal and informal institutional framework) and ecosystems (EC) (e.g. vaccine production ecosystem and supporting ecosystems such as mask production ecosystem) according to their vision and capabilities. In addition, this environment defines multi-level boundaries between biomes and ecosystems; for example, the actors related to the WHO (ET) operate on the border between the vaccine discovery knowledge ecosystem (including scientists, research centres and universities) and the vaccine business ecosystem (including vaccine manufacturers and exporters).

In other words, national innovation biomes (BI) [e.g. European Union (EU), Food and Drug Administration (FDA), United States Department of Agriculture (USDA), House of Representatives and Senate, Department of Health and Human Services (HHS), Center for Disease Control and Prevention (CDC)], innovation ecotones [e.g. African Development Bank (AfDB) as a leading player not only in providing financial support but also technical support to help African governments adopt better development policies] and the knowledge and technological systems (EC) under the innovation biosphere of COVID-19 that develop innovative solutions, facilitate academia-industry-government collaborations, create new job opportunities, enhance existing industries and improve the economy and society. For example, manufacturers collaborate with universities and research centres to produce high-quality medical masks and gloves using new knowledge and technologies. These activities are defined under innovation ecotones (ET) in the COVID-19 innovation biosphere. In knowledge and technological ecosystems, the collaboration between technology firms and research centres aims to develop innovative technologies to combat COVID-19. For example, IT firms and research centres in the United States collaborated to develop intelligent systems to prevent and manage COVID-19. These systems include prevention, treatment and monitoring applications; smart diagnostic and treatment systems; and smart medical devices [8, 16, 49]. In another example, technological ecosystems develop social solutions for COVID-19 consequences through public–non-governmental organization (NGO) collaboration (RL). In developing countries, public agencies, social activists and individuals provide health services and psychological support, as well as distribute food, medical masks and gloves to reduce the adverse effects of COVID-19 on society.

### Macro level

At the macro level, the value network (NE) of the innovation biosphere of COVID-19 includes researchers and scientists as the human resources, governments and firms (e.g. Pfizer, AstraZeneca, Johnson & Johnson and Moderna) as financial and technological resources, universities and research institutes (e.g. Harvard, Oxford and Stanford) as knowledge resources, manufacturing firms including 3M, Honeywell and Walmart (production of medical equipment) and FedEx, UPS, Uber and Lyft (transportation of medical equipment and COVID-19 vaccines) as infrastructure resources [57] (RE). In addition, mission-oriented innovation policies (MI) were utilized to create joint opportunities using communication technologies to determine responsibilities and authorities and coordinate strategies and as an organizing and adapting system (CO) with changing management approaches; new technologies to improve organizational capabilities and coordinate with other systems (FL) in formulating joint strategies; and collected data and risk analysis, which has been developed under conditions of uncertainty towards the common goal of creating and developing new solutions for COVID-19. The most obvious example of this feature is the production of the COVID-19 vaccine and the arrangements for its fair and widespread distribution in all countries [53].

At this level, one of the features of the innovation biosphere of COVID-19 was the reinforcement of technological infrastructure (TE), including diagnostic tools [e.g. polymerase chain reaction (PCR), antigenic rapid tests and serology] [32, 43], modern technologies (e.g. machine learning, neural networks, AI and data mining) [17, 31, 42, 47], health data (data of patients, tests, vaccines and treatments for analysis, assessment, and identification of strengths and weaknesses), online platforms (communication and collaboration between innovators at the global level, transfer of health data, and participation in R&D to develop innovative solutions) [22, 63], medical equipment and communication networks. Due to these infrastructures, numerous relationships (IN) have been formed between government policies and laws; institutions such as the WHO, World Trade Organization, World Bank and organizations related to R&D; as well as collaboration between countries and between universities and industries, which provide financial and credit resources for developing the academic and scientific community and scientists for R&D in the direction of covering and managing the innovation biosphere of COVID-19.

**Fig. 6:**
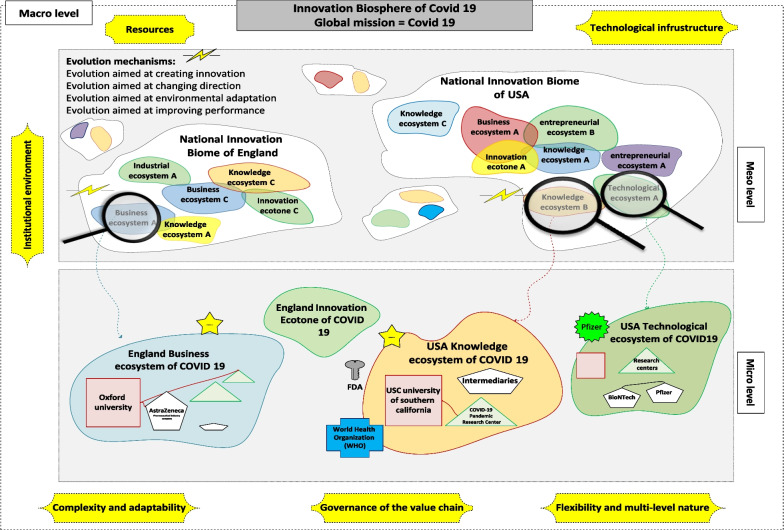
The three-level framework of innovation biosphere of Covid-19 and the connections between them along with some examples (self-compilation)

In general, the analysis of the data related to the case study of this research, namely, COVID-19, shows that the biosphere approach of innovation can be used as an analytical framework for mapping global cooperation, especially regarding global health challenges. It also helps in the coordinated management of global activities. This important issue has not been considered by adapting ecological features such as the metaphor of the biosphere. The three-level framework (macro, meso, micro) in this approach provides the possibility of identifying and analysing all actors at different levels under the global health mission. Therefore, innovation biosphere helps to understand the necessity of knowledge flow and co-evolution between actors at micro, meso and macro levels in the field of health. All in all, the effect of global collaboration and co-evolution of different actors and subsystems in facing the COVID-19 grand challenge with an eco-systemic and eco-innovation approach was evident, which subsequently led to the end of the pandemic after 4 years. Based on what was mentioned above, the effect of global cooperation, role-creation and co-evolution of different actors and subsystems in facing the challenge of Covid-19 under a systemic and eco-innovation approach has been evident. This subsequently led to the cessation of the pandemic after about four years. Figure 6 tries to provide a general and multi-level view of the proposed framework for managing the grand challenge of COVID-19 (along with some examples that were mentioned earlier) and explain the connections between the three levels. We have called it the innovation biosphere of COVID-19. In each of the mentioned levels, some examples have been presented to make the understanding of the mentioned dynamics in the innovation management process clearer in the face of COVID-19. At the macro level, it has shown a general map along with the main features of the macro-framework of the innovation biosphere of COVID-19; As shown, by considering COVID-19 as a global mission (as one of the characteristics listed at the macro level in accordance with the conceptual framework of the innovation biosphere), an orientation to the main pillars of the innovation biosphere has been formed. At the meso level, some examples of the types of ecosystems, ecotones, and active innovation biomes in the innovation biosphere of Covid-19 have been shown, and for this purpose, examples related to the countries of United States and England have been used. Finally, at the micro level, the interactions between some general and specific actors in the innovation biosphere of COVID-19 are shown under the respective ecosystems. It is worth mentioning that Fig. 6 is only for a better understanding of the framework proposed in this research and many actors are active in the innovation biosphere of COVID-19 and it is limited to presenting some examples.

## Conclusions

Globalization and growing communications have caused many grand challenges that did not emerge, at least globally, until one or two centuries ago. However, today the world witnesses various global crises, the most important of which are climate change and global warming. Some national and regional crises can quickly become grand global challenges; for instance, the Russia–Ukraine war caused a global food and energy crisis within a few weeks, and economic collapse in the Middle East and Africa led to numerous waves of immigration towards Europe.

So what were the characteristics of the COVID-19 pandemic that led to global collaboration, and why did such a unified effort not happen in the case of other grand challenges (e.g. lack of fresh water in many countries leading to economic collapse and civil and international wars)? Indeed, the unique characteristics of COVID-19, including the rapid global outbreak, the shutdown of major economic sectors worldwide and the public panic, were crucial. However, it was clear from the beginning that social methods (e.g. quarantine or herd safety) would not suffice, with science and technology as the leading element to end the crisis. In the following, two categories of implications are presented: implications for managing possible future pandemics by analysing the case with the innovation biosphere lens and the implications of the global encounter with COVID-19 for enhancing the global innovation biosphere metaphor.

### Implications of the innovation biosphere framework for managing grand health challenges

Focussing on the critical features of the innovation biosphere framework obtained from the literature review, the most crucial policy implications for managing grand health challenges are:

- **Role of self-organization:** Governments normally are expected to act to solve crises, but they can hardly reach an agreement when global crises emerge. For example, effective agreements have yet to be reached to eliminate poverty or climate change. Networking between universities, scientific organizations and NGOs while enriching the role of international organizations (e.g. WHO) will lead to self-organization in the innovation biosphere of grand global challenges.
- **Capability of global value networks**: With innovative activities such as R&D, production and distribution of products and services at the innovation biosphere level and beyond national innovation biomes, the costs will decline while the consumers’ access enhances. Therefore, removing tariff and non-tariff barriers between countries in the health field will help solve global health crises.
- **Mission orientation**: The development of health innovation is interdisciplinary; however, some countries with specific institutional structures and non-integrated and non-coherent financing channels manage this field through intra-sectoral policies. These traditional and isolated policies are grand challenges for coordinating efforts towards the goals and strategic missions of the national health sector. Therefore, better coordination and orientation of health policies based on the new generation of mission-oriented (not functional) innovation policies are vital, as the COVID-19 pandemic management has verified the need for such transformation. These characteristics also reflect in the mission-oriented and challenge-driven aspects of the innovation biosphere.
- **Co-evolution between actors**: In natural ecosystems as subsets of the biosphere, co-evolution between actors resulting from collaboration or competition is crucial. Thus, knowledge flow must ensure that actors co-evolve in the health field’s micro, meso, and macro levels.

### Implications of COVID-19 grand challenge to enhance innovation biosphere metaphor

In this section, highlighting the critical lessons from the COVID-19 pandemic and the findings of the article, the most noteworthy theoretical implications for strengthening the proposed innovation biosphere framework – also applicable in other global missions and challenges – are presented as follows:

- **Global cohesion**: Despite diagnosing main corona variants mostly in the South (Alpha in China and Beta and Omicron in South Africa), effective vaccines were often developed in the North. Repeating the acquired immunodeficiency syndrome (AIDS) drug experience would have the South countries caught up in the pandemic for years, threatening the North as well. Understanding this global cohesion led the North to help the South, and the same approach is crucial for other grand challenges.
- **Oligopoly market**: Various research suggested that technology development emerges better in oligopoly markets (with limited competition) compared with fully competitive or monopolistic markets. The development of corona vaccine in limited but multiple competitions proved the validity of this hypothesis.
- **Supporting local innovations**: A significant proportion of human knowledge has been realized through local knowledge and innovations. During the pandemic, these innovators hardly tried contributing to the dominant flow of global innovation. The innovation biosphere should support developing and disseminating these innovations, as solutions developed in one context (e.g. dealing with water shortage) can be beneficial in other contexts.
- **Critical role of basic research**: The debate about whether basic research budgets should be limited is ongoing in various developing countries. The corona pandemic case indicated that this type of research mainly solves grand challenges, although other biosphere components should also contribute.
- **Deregulation**: Considering the current regulations regarding the testing of drugs and vaccines, the development of the corona vaccine would take years. The innovation biosphere of grand challenges must thus bypass these kinds of regulations.

Learning from the COVID-19 pandemic, science, technology and innovation should move towards developing sustainable, justice-oriented and resilient societies. Therefore, governments should design science, technology and innovation policies to fulfil the realization of expected systems and societies with the above characteristics. The proposed innovation biosphere framework can serve as a basis for analysing and formulating such policies. The innovation biosphere is an idea presented in this article for the first time. Therefore, future studies can analyse and develop its conceptual framework to address grand global challenges better.

## Data Availability

The datasets used and analysed during the current study are available from the corresponding author on reasonable request.

## Footnotes

1 https://www.worldometers.info/coronavirus.

2 This article introduces the innovation biosphere as a macro-metaphor and its constituent components as related sub-metaphors.

3 In ecology, the biosphere is the actual or potentially habitable places on the Earth that have life or the potential for life, including a collection of biomes and ecosystems [38]. The biosphere has two main characteristics: (1) it is a very complex and adaptable system that contains many diverse organisms (millions of animal species and hundreds of thousands of plant species), and the existence of such diversity is the product of a long evolutionary process; and (2) with a mosaic structure irregular geometry, biosphere includes a chaotic distribution of inanimate elements (uneven expansion oceans, continents, mountain ranges, rivers and deserts) and animate elements (heterogeneous distribution of living organisms in polar or arid regions or the heights and depths of the deep seas compared with temperate and tropical zones).

4 https://devex.shorthandstories.com/key-actors--who-is-leading-the-covid-19-aid-response-/index.html.
